# Effects of physical and mental health factors and family function on the self‐perception of aging in the elderly of Chinese community

**DOI:** 10.1002/brb3.2528

**Published:** 2022-08-03

**Authors:** Fengying Gao, Lijuan Zhou, Ya Gao, Yinglong Zhang, Aifang Zuo, Xinkun Zhang

**Affiliations:** ^1^ Department of Seven Orthopaedics ‐ trauma orthopaedics Handan Central Hospital of Hebei Province of China Handan Hebei Province China; ^2^ Department of Gynaecology and Obstetrics Weixian People's Hospital of Handan City in Hebei Province of China Weixian Hebei Province China; ^3^ Department of Management Section Hebei Iron and Steel Group Cold rolling plant of Handan Iron and Steel Company Shijiazhuang Hebei Province China

**Keywords:** elderly, family functions, physical and mental health, self‐perceptions of ageing

## Abstract

**Background:**

To examine the effects of physical and mental health factors and family functioning on the self‐perception of ageing in elderly people.

**Methods:**

A random cluster sampling method was used to select elderly people aged over 60 from three communities in Handan City. Subjects were evaluated via face‐to‐face interviews using the Chinese version of the Ageing Perception Questionnaire, the Family Function Scale, the SF‐36 Short‐Form Health Survey, and a self‐compiled general questionnaire. A single factor and stepwise multiple regression analysis were evaluated using SPSS 17.0 software.

**Results:**

Among the 1815 elderly people surveyed, the total negative dimension score was 91.67 ± 16.58 with an index of 73.34%, which is higher than the positive dimension score (6.01 ± 0.52, 60.10%). Elderly people with varying degrees of family dysfunction accounted for 11.63%, and the score for self‐perceived ageing in elderly participants with good family function was 95.74 ± 12.63. The proportions with poor physical and mental health factors were 45.40% and 28.10%, respectively, and the scores for ageing self‐perception in elderly participants with good or moderate mental health were 89.11 ± 12.65 and 86.22 ± 12.58, respectively. A stepwise multiple regression analysis showed that age, presence of a spouse, and family function were positive protective factors for ageing self‐perception, while physical health factors were risk factors for the positive dimension of self‐perceived ageing. Age and family function were risk factors for the negative dimension of ageing self‐perception, while physical and mental health factors were protective factors for the negative dimension of self‐perceived ageing.

**Conclusions:**

Younger elderly and elderly people with good family function have positive self‐perceptions of ageing, while elderly participants with poor physical and mental health have a negative perception of ageing.

## Graphical abstract

The young old and elderly with better family function were more positive on self‐perception of aging, while the elderly with poor physical and mental health have a negative perception of aging.

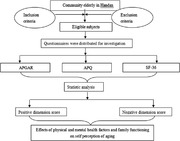

## 1 BACKGROUND

Elderly adults are prone to various chronic diseases as they age; however, the impacts of diseases on health outcomes will vary, as self‐perceived ageing is an important influential factor (Levy & Bavishi, 2018). Based on the self‐adjustment model, Professor Barker (Barker et al., 2007) defined self‐perceived ageing as the subjective perceptions and emotional reactions of elderly adults when faced with the psychological, physical, and social impacts of ageing. According to these different views, ageing can be perceived as either positive or negative. Elderly adults in a positive ageing state are more likely to adopt preventative health behaviors, including a balanced diet, exercise, and compliance with medication requirements. Older adults in a negative ageing state tend to refuse life‐prolonging medical interventions, which diminishes their will to live and affects both their life expectancy and quality of life.

A study with a 4‐year follow‐up showed the impact on cognitive functioning and found that people with negative views of ageing were more likely to develop dementia than those with positive outlooks (Levy et al., 2018). The study also found that the life expectancy of those in a positive ageing state increased by 7.5 years compared with those with a negative perspective (Levy et al., 2018; Schroyen et al., 2017; Sun et al., 2017). Stewart et al. (2012) found that the mortality rate of elderly people with a negative perception of ageing was more than twice that of elderly people with a positive perception.

China has one of the largest populations of elderly people in the world, and the physical and mental health of its elderly citizens has attracted much attention. The early detection of and intervention in self‐perceived ageing are important for prolonging life expectancy, improving quality of life and reducing social and family burdens. Therefore, the present study investigated the effects of family function and physical and mental health on self‐perceived ageing in people over the age of 60 in Handan City, Hebei Province, China.

## 2 METHODS

### 2.1 Subjects

A randomized cluster sampling method was used. The 312 communities in the main urban area of Handan City (Congtai, Fuxing, and Hanshan districts) were numbered from 1 to 312, and three communities were randomly selected: the Asia–Pacific and Guangxi community, the Huayuan second community and the Baileyuan community. From those communities, a total of 1876 elderly adults aged over 60 years were evaluated. The inclusion criteria for participation in this study were as follows: (1) age ≥ 60 years, (2) resident for ≥6 months, and (3) informed consent given. The exclusion criteria were the presence of the following: (1) a previous history of mental illness, (2) an expression or communication disorder, and (3) a discrepancy between registered and actual residences. The first two exclusion criteria were specified mainly due to the inability of older adults with mental illnesses or language difficulties to express themselves properly and fill out the scales, which would bias the results. The third criteria excluded people who did not live in the selected communities to ensure the homogeneity of the study population and to avoid the influence of different places of residence on the results. A total of 1815 participants were evaluated, and the response rate was 96.7%.

### 2.2 Methods

The study was conducted between June and November 2017 and involved a face‐to‐face interview‐based survey with a standardized questionnaire. All the subjects gave written informed consent, and a household questionnaire was provided for those who could not attend the survey location in person. Information was collected under the following four categories: (1) general information, including age, gender, marital status, education background, and economic income. (2) The self‐perception of ageing, which used Professor Barker's Ageing Perception Questionnaire (translated by Hu, 2013). The Cronbach's alpha (α) coefficient of the scale was .884. The positive dimensions included the two subdimensions of positive control and positive consequences, while the negative dimensions included the following six subdimensions: identity, timeline chronic, timeline cyclical, emotional representations, negative consequences, and negative control. There were 46 items in total, and the “Yes/No” subdimension scored “Yes” as 1 and “No” as 0. The score of this subdimension equaled the total score of health problems caused by ageing divided by the total score of health problems multiplied by 100. The other seven subdimensions were scored on a five‐point Likert scale. Four items in the negative control dimension were reverse‐scoring items, and the score of each subdimension was the average of the total score of each subdimension. A high score in the positive dimension indicated a more positive self‐perception of ageing, while a high score in the negative dimension signified a more negative view. (3) Family function: the evaluation used the APGAR score, which was designed in the United States by Dr. Smilkstein (Smilkstein et al., 1982; Zhang, 2005). It was translated into Chinese and revised by Zhang Zuoji with good internal consistency. The Cronbach's α coefficient was .748, and the evaluation included five items, each of which was scored on three levels. The total score range was between 0 and 10. Scores between 0 and 3 indicated severe family dysfunction, 4 to 6 indicated moderate family dysfunction, and 7 to 10 indicated good family function. The higher the total score, the better the family function. (4) Physical and mental health: The medical outcome survey from the SF‐36 Short‐Form Health Survey (developed by the Institute of Health Education in Boston, USA) was used in the assessment (Guillemin et al., 1993). It included comprehensive evaluations of physical health (encompassing the four dimensions of physical functioning, physical role, bodily pain, and general health) and mental health (encompassing the four dimensions of vitality, social functioning, emotional role, and mental health). The original score was obtained using the hundred‐mark system, which was converted using the standardized scoring method in which scores of 0, 60, and 85 indicated poor, moderate, and good health, respectively. A higher score indicated a better health status.

#### 2.2.1 Technical roadmap

**FIGURE 1:**
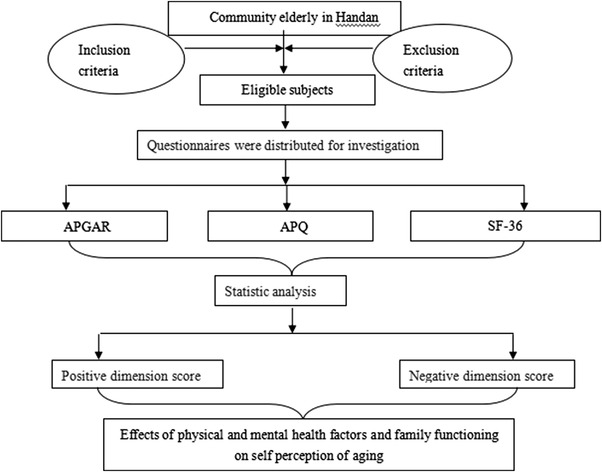
The flow chart

The information can be seen in Figure 1.

### 2.3 Statistical analysis

A database was created using Excel, and SPSS 17.0 software was used for the statistical analysis. Continuous variables were presented as mean ± standard deviation, while categorical variables were presented as counts (percentages). To examine the effects of general information, physical factors and mental factors on the self‐perception of ageing, a *t* test or an analysis of variance was used initially for the univariable analysis, and a stepwise multiple regression analysis was used at the multivariable level to exclude potential confounding factors.

## 3 RESULTS

### 3.1 General demographics

Among the 1815 elderly people surveyed, 712 (39.22%) were male and 1103 (60.77%) were female. The participants were aged between 60 and 95 years (average: 70.00 years). Of them, 949 (52.629%) were aged between 60 and 69 years, 575 (31.68%) were aged between 70 and 79, and 291 (16.03%) were aged over 80 years. A total of 1413 (77.85%) subjects had partners, 402 (22.15%) were widowed, 568 (31.29%) lived with their children, 1120 (61.761%) lived with their partners and 127 (7.00%) lived alone. There were 1608 (88.60%) people with a junior high school education or below and 207 (11.40%) with a high school education or above. A total of 440 (24.24%) were professionals, 940 (51.79%) were workers, and 435 (23.97%) were in the “other” category. A total of 667 (36.74%) elderly people had a main monthly income of <2000 yuan, while 1148 (63.25%) had a monthly income of 2000 yuan or more. Finally, 1353 (74.55%) participants had a chronic disease, while 462 people (25.50%) had none.

### 3.2 Scores for physical/mental health factors and family function

The overall physical health score was 60.79 ± 12.42, which was in the middle and lower levels. Of the 1815 participants, 824 (45.40%) were in poor physical health and scored 39.85 ± 7.45. The overall score for mental health was 68.21 ± 10.24, which was at the medium level. Of the total 1815 participants, 510 (28.10%) had poor mental health with a score of 45.96 ± 9.38. The total score for family function was 7.95 ± 1.36, and 11.63% (211 out of 1815) of the elderly participants had different degrees of family dysfunction, scoring 5.63 ± 0.86.

### 3.3 The effects of general information on self‐perceived ageing

1. The effects of general information on the total score for the positive dimension of self‐perceived ageing are presented in Table 1. Among the 1815 subjects interviewed, the total score for the positive dimension was 6.01 ± 0.52 with a score index of 60.10%. Elderly adults who were older, spouseless, living alone and with a low economic income had lower positive dimension scores (*p* < .05). Gender, education, and preretirement occupation did not affect the positive dimension scores.
2. The effect of general information on the total score for the negative dimension of self‐perceived ageing is given in Table 2. The total negative dimension score was 91.67 ± 16.58 with a score index of 73.34%, which was higher than that of the positive dimension. The negative dimension scores were higher for adults who were elderly, who had no spouse, who lived alone, who had received higher education, and who had other occupations before retiring (*p* < .05). Gender, education, and preretirement occupation did not affect the positive dimension scores. Neither gender nor economic income affected the scores for the negative dimension.

**TABLE 1:** Effect of general information on the total score of positive dimension of self‐perception of aging (*n* = 1815, $\bar{x}$±s)

Characteristic	Case (*n*)	Positive dimension score	*F*	*p*
Age^1^ (years)				
∼60	949	6.14±0.51	67.231	<.001
∼70	575	6.02±0.49		
80∼95	291	5.53±0.38		
Gender				
Male	712	5.82±0.79	−1.570*	.117
Female	1103	5.88±0.77		
Spouse				
Yes	1413	6.10±0.80	−8.854*	.001
No	402	5.69±0.67		
Living status^2^				
Living with children	568	5.93±0.78	13.543	<.001
Living with spouse	1120	5.92±0.76		
Living alone	127	5.54±0.78		
Education^3^				
Illiterate	982	5.90±0.75	1.078	.341
Primary/junior high school	626	5.86±0.60		
High school and above	207	5.93±0.64		
Occupation before retirement^4^				
Cadre	440	5.90±0.72	0.264	.768
Worker	940	5.92±0.70		
Other	435	5.89±0.57		
Economic income^5^ (yuan/month)				
<1000	285	5.80±0.63	6.121	<.001
∼1000	382	5.87±0.54		
∼2000	922	5.92±0.57		
3000∼6000	226	6.09±0.62		

* *t* Value.

^1^ Trend *F* value was 134.154, *p* < .001.

^2^ Trend *F* value was 24.617, *p* < .001.

^3^ Trend *F* value was 0.374, *p* = .541.

^4^ Trend *F* value was 0.234, *p* = .629.

^5^ Trend *F* value was 13.604, *p* < .001.

**TABLE 2:** Effect of general information on the total score of negative dimension of self‐perception of aging (*n* = 1815, $\bar{x}$±s)

Characteristic	Case (*n*)	Negative dimension score	*F*	*p*
Age^1^ (years)				
∼60	949	86.45±11.27	74.997	<.001
∼70	575	92.57±12.65		
80∼97	291	100.64±10.87		
Gender				
Male	712	91.86±19.07	0.459*	.647
Female	1103	91.45±18.28		
Spouse				
Yes	1413	87.04±18.34	−8.070*	<.001
No	402	94.16±13.38		
Living status^2^				
Living with children	568	93.31±13.02	13.843	<.001
Living with spouse	1120	88.89±12.24		
Living alone	127	94.55±11.71		
Education^3^				
Illiterate	982	92.17±16.34	8.022	<.001
Primary/junior high school	626	90.76±16.45		
High school and above	207	87.64±16.98		
Occupation before retirement^4^				
Cadre	440	87.66±16.61	9.186	<.001
Worker	940	91.04±14.93		
Other	435	92.90±17.00		
Economic income^5^ (yuan/month)				
<1000	285	93.28±13.83	2.567	.053
∼1000	382	90.26±12.94		
∼2000	922	89.88±12.79		
3000∼6000	226	91.25±14.12		

* *t* Value.

^1^ Trend *F* value was 140.441.759, *p* < .001.

^2^ Trend *F* value was 0.472, *p* = .492.

^3^ Trend *F* value was 16.022, *p* < .001.

^4^ Trend *F* value was 17.579, *p* < .001.

^5^ Trend *F* value was 1.622, *p* = .203.

### 3.4 The effects of physical/mental health factors and family function on self‐perceived ageing

1. The effects of physical/mental health factors and family function on the total positive dimension score for self‐perceived ageing are given in Table 3. The poorer the physical/mental health factors and the more family dysfunctions, the lower the total positive dimension score (*p* < .05).
2. The effects of physical/mental health factors and family function on the total negative dimension score for the self‐perception of ageing are presented in Table 4. The poorer the physical and mental health factors and the more family dysfunctions, the higher the total negative dimension score (*p* < .05).

**TABLE 3:** Effect of physical and mental health factors and family functioning on the total score of positive dimension of self‐perception of aging (*n* = 1815, $\bar{x}$±s)

Items	Case (*n*)	Positive dimension score	*F*	*p*
Family functioning^1^				
Good family functioning	1604	5.98±0.39	62.468	<.001
Moderate family dysfunction	122	5.36±0.28		
Severe family dysfunction	89	4.04±0.13		
Physical health factors^2^				
Good	311	6.21±0.86	35.609	<.001
Moderate	680	5.97±0.76		
Poor	824	5.78±0.78		
Mental health factors^3^				
Good	224	6.09±0.83	15.105	<.001
Moderate	1081	5.96±0.75		
Poor	510	5.77±0.82		

^1^ Trend *F* value was 55.114, *p* < .001.

^2^ Trend *F* value was 67.552, *p* < .001.

^3^ Trend *F* value was 11.426, *p* < .001.

**TABLE 4:** Effect of physical and mental health factors and family functioning on the total score of negative dimension of self‐perception of aging (*n* = 1815, $\bar{x}$±s)

Items	Case (*n*)	Negative dimension score	*F*	*p*
Family functioning^1^				
Good family functioning	1604	89.92±10.83	19.451	<.001
Moderate family dysfunction	122	100.15±9.68		
Severe family dysfunction	89	102.47±7.84		
Physical health factors^2^				
Good	311	83.33±10.06	77.204	<.001
Moderate	680	87.35±12.62		
Poor	824	96.17±11.53		
Mental health factors^3^				
Good	224	80.52±10.81	82.391	<.001
Moderate	1081	89.32±11.05		
Poor	510	97.97±12.14		

^1^ Trend *F* value is 4.168, *p* = .041.

^2^ Trend *F* value is 116.934, *p* < .001.

^3^ Trend *F* value is 149.573, *p* < .001.

### 3.5 The effects of physical/mental health factors and family function on the subdimension of self‐perceived ageing

1. The effects of physical health factors on the subdimension of self‐perceived ageing are presented in Table 5. Older adults with good physical health factors scored higher for each positive dimension than those with moderate or poor physical health factors, while they scored lower than those with moderate or poor physical health factors (*p* < .05) for each negative dimension.
2. The effects of mental health factors on the subdimension of self‐perceived ageing are shown in Table 6. Older adults with moderate or poor mental health factors scored higher for the negative subdimension and lower for the positive subdimension compared with those with good mental health factors (*p* < .05).
3. The effect of family function on the subdimension of self‐perceived ageing is presented in Table 7. Except for negative dimension consequences, older adults with family dysfunctions scored higher for all negative subdimensions and lower for the positive subdimension compared with those with a good level of family function (*p* < .05).

**TABLE 5:** Effects of physical health factors on subdimension of self‐perception of aging (*n* = 1815, $\bar{x}$±s)

	Physical health factors				
Self‐perception of aging	Good	Moderate	Poor	*F*	*p*
Identity	69.88±12.64	73.35±11.38	80.54±10.68	34.573*	<.001
Timeline chronic	2.77±0.93	3.08±0.93	3.34±0.84	46.807*	<.001
Timeline cyclical	2.59±0.56	2.59±0.61	2.62±0.61	9.534	<.001
Emotional representations	2.46±0.58	2.58±0.59	2.74±0.56	31.911	<.001
Control negative	2.78±0.45	2.84±0.49	3.09±0.46	81.503*	<.001
Consequences negative	2.85±0.77	2.87±0.72	3.13±0.59	31.876*	<.001
Consequences positive	2.88±0.70	2.90±0.69	2.72±0.69	14.811	<.001
Control positive	3.41±0.53	3.26±0.53	3.06±0.59	51.631*	<.001

* The normal variance is not uniform and the approximate *F* value is taken.

**TABLE 6:** Effects of mental health factors on subdimension of self‐perception of aging (*n* = 1815, $\bar{x}$±s)

	Physical health factors				
Self‐perception of aging	Good	Moderate	Poor	*F*	*p*
Identity	67.19±12.50	75.12±10.69	81.81±11.51	38.383*	<.001
Timeline chronic	2.90±0.73	3.12±0.74	3.79±0.81	17.256*	<.001
Timeline cyclical	2.68±0.56	2.60±0.82	2.83±0.62	9.221	<.001
Emotional representations	2.52±0.60	2.58±0.57	2.87±0.61	26.974*	<.001
Control negative	2.67±0.48	2.89±0.50	3.25±0.47	156.535	<.001
Consequences negative	2.55±0.74	3.01±0.70	3.41±0.68	52.935*	<.001
Consequences positive	2.88±0.58	2.86±0.50	2.69±0.57	12.627	<.001
Control positive	3.37±0.52	3.23±0.54	3.02±0.64	33.191*	<.001

* The normal variance is not uniform and the approximate *F* value is taken.

**TABLE 7:** Effect of family functioning on subdimension of self‐perception of aging (*n* = 1815, $\bar{x}$±s)

Self‐perception of aging	Good family functioning (1604)	Family dysfunction (211)	*T*	*p*
Identity	75.40±12.26	84.03±10.62	−8.392^△^	<.001
Timeline chronic	3.14±0.92	3.24±0.76	−1.455^△^	.148
Timeline cyclical	2.64±0.59	2.83±0.65	−3.288	.001
Emotional representations	2.61±0.58	2.90±0.51	−6.251^△^	<.001
Control negative	2.93±0.41	3.16±0.39	−6.485	<.001
Consequences negative	3.22±0.68	3.12±0.57	1.905	.059
Consequences positive	2.85±0.60	2.33±0.54	−10.454	<.001
Control positive	3.22±0.56	2.85±0.74	−5.525^△^	<.001

^△^ The normal variance is not uniform, and the approximate *t*‐value is taken.

### 3.6 Multifactor analysis of self‐perceived ageing

1. The multifactor analysis of the positive dimensions of the self‐perception of ageing is presented in Table 8. The total score of the positive dimensions of self‐perception was used as the dependent variable, and the variables with statistically significant differences in the univariate analysis were entered gradually into the equation as independent variables for the linear multiple regression analysis. The results showed that age, spouse, and family function were protective factors, while physical health issues were risk factors for the positive dimension of self‐perceived ageing.
2. The multifactor analysis of the negative dimensions of self‐perceived ageing is provided in Table 9. The total score of the negative dimensions of self‐perceived ageing was used as the dependent variable, and the variables with statistically significant differences in the univariate analysis were entered gradually into the equation as independent variables for the linear multiple regression analysis. The results showed that age and family function were risk factors, while physical and mental health issues were protective factors for the negative dimension of self‐perceived ageing.

**TABLE 8:** Multiple linear regression analysis of the positive dimensions of self‐perception of aging

Model	Nonstandardized regression coefficient	Standard error	Standardized regression coefficient	*t*	*p*
Constant	5.919	0.103	—	57.355	<.001
Age	−0.154	0.027	−0.141	−5.805	<.001
Spouse	−0.145	0.047	−0.074	−3.066	.002
Family functioning	−0.639	0.065	−0.223	−9.863	<.001
Physical health	0.169	0.025	0.154	6.697	<.001

**TABLE 9:** Multiple linear regression analysis of the negative dimensions of self‐perception of aging

Model	Nonstandardized regression coefficient	Standard error	Standardized regression coefficient	*t*	*p*
Constant	92.140	2.647	—	34.458	<.001
Age	4.875	0.577	0.195	8.443	<.001
Spouse	3.154	1.494	0.048	2.111	.035
Family functioning	−2.984	0.733	−0.119	−4.072	<.001
Physical health	−4.196	0.898	−0.139	−4.672	<.001
Mental heath	92.140	2.647	–	34.458	<.001

## 4 DISCUSSION AND CONCLUSIONS

The present study examined the effects of physical and mental health factors and family function on the self‐perception of ageing in a relatively large cohort of elderly adults. The main findings can be summarized as follows: (1) The total negative dimension score was 91.67 ± 16.58 with an index of 73.34%, which is higher than that of the positive dimension (6.01 ± 0.52, 60.10%). Elderly adults who were older, spouseless, living alone, and with a low economic income had lower positive dimension scores. (2) Physical/mental health factors were negatively associated with the total negative dimension score. (3) The multivariable analysis revealed that age, spouse, and family function were positive protective factors for the self‐perception of ageing, while physical health factors were risk factors for its positive dimensions. Age and family function were risk factors for the negative dimensions of self‐perceived ageing, and physical and mental health factors were protective factors for its negative dimensions.

### 4.1 The effects of general information on self‐perceived ageing

This study showed that age was a common influential factor for both the positive and negative dimensions of self‐perceived ageing. The younger the participant, the more positive their self‐perceived ageing, while the older the person, the more negative the perception. The results of the multifactor analysis confirmed that age was an additional influential factor; the older participants had more pronounced self‐perceived ageing, which is consistent with other related findings (Martin et al., 2015). With increasing age, older adults gradually reduced or even reversed their functional roles in their families and society. They experienced greater psychological disparities, believed that they could not control the ageing process, and were more inclined to experience the negative emotional effects of ageing.

Declining physical function, poor independent living ability, and increased dependence on family and society prompted participants to become concerned with their changes, and even slight variations in health status were perceived clearly. Furthermore, different occupations and work experiences affected the understanding and feelings of the elderly participants. When changes, such as ageing, occur in the body, people can perceive them and respond rapidly, reducing the perception time and taking appropriate measures to cope with the resulting changes.

The main reason for the negative self‐perception of ageing among people without spouses compared with those with partners is that older adults with spouses provide care and emotional support for each other. They are more likely to maintain strong and positive emotional and social states (Martin et al., 2015) and have more positive perceptions of ageing. Conversely, older adults without spouses who are prone to negative emotions, such as loneliness, and who participate in fewer social activities are more likely to attribute their existing health problems to growing older and experience more negative feelings about ageing.

Older people living alone are often lonely and isolated in their daily lives. Without a partner, their lack of interaction means that when they experience anxiety, depression, or other psychological difficulties, intervention comes too late; they become depressed over time, and the occurrence of illnesses worsens their depression and mental health symptoms. Depression adversely affects the ageing of older people and forms a vicious circle that increases the negative perception of becoming older. Therefore, it is suggested that more attention should be paid to older people who are widowed and living alone. There should be a focus on providing ageing‐related education and implementing various measures to help establish a positive ageing perspective to enable people to transition through the ageing process.

The effect of gender on the self‐perception of ageing was not observed in this study. This differs from the results of overseas research (Tung et al., 2012) and could be attributed to geographical variations and differences in study subjects.

### 4.2 The effects of physical/mental health factors on self‐perceived ageing

Levy et al. (2008) showed that patients with cardiovascular disease have a more negative self‐perception of ageing due to physical reasons. Additionally, they confirmed that negative ageing states act as direct stressors that increase cardiovascular stresses and strains and negatively impact the recovery of patients with myocardial infarction. This study also showed that a person's physical health status is an important influential factor on the self‐perception of ageing; this is consistent with other studies (e.g., Schroyen et al., 2017; Wurm et al., 2014) and could be related to poorer physical health, limited outdoor exercise and depression in older adults.

Studies have found that older adults with poor mental health have a more negative self‐perception of ageing. Zhao et al.’s (2017) study confirmed that older adults with self‐assessed loneliness, depression, and poor health have a more negative self‐perception of ageing. Furthermore, a state of negative ageing exacerbates psychological symptoms, including depression (Kwak et al., 2014; Levy et al., 2014b), forming a vicious circle that seriously affects the quality of life of older adults.

However, the ageing state is not completely irreversible (Levy et al., 2014a), as demonstrated by Dekeyser et al. (2008), who conducted attention training for elderly people. The participants became active and energetic, and their physical functioning, psychological condition, and social participation all improved, suggesting that the early detection of and intervention in a negative ageing state are critical.

### 4.3 Effects of family function on self‐perceived ageing

The results of the present study demonstrated that family function influences the self‐perception of ageing among older adults. Elderly people with good family function receive more satisfactory spiritual comfort and daily care from their spouses and children. The resulting positive spiritual and life status has a positive effect on growing older and helps to create a more positive self‐perception of ageing (Kaspar et al., 2021; Plys & Smith, 2021). Conversely, older adults with family dysfunctions have a more negative self‐perception of ageing. The family is the most basic social unit; it is related closely to the life and health of elderly people and provides their main spiritual and psychological support (Bode et al., 2012). If older people receive appropriate care and support from their families, they will be able to negotiate the ageing process successfully and maintain their physical and mental health by developing a positive self‐perception of ageing. Several studies have confirmed that older adults who live with their families and have frequent contact with them perceive ageing more positively (Hu, 2013; Iannitti & Palmieri, 2011). Therefore, it is recommended that older adults who live alone should reside with their children and interact frequently with them, while community health care workers should publicize the importance of emotional comfort and support for older adults to promote the positive management of ageing and improve their quality of life.

## 5 RESEARCH SHORTCOMINGS AND PROSPECTS

This study involved only a limited number of communities in Handan City and analyzed and explored only the effects of family function and physical/mental health factors on self‐perceived ageing. Due to geographical limitations, caution is needed as to whether the conclusions drawn from this study can be applied to other areas. Considering the principle of medical ethics, the subjects participated voluntarily, but as they may have been biased, the influence of these factors should be considered in the generalization and application of the results. Additionally, this study adopted a cross‐sectional design and analyzed only the influence of physical/mental health factors and family function on self‐perceived ageing at a certain point in time; therefore, caution is needed in inferring the causal relationship between the influential factors and self‐perceived ageing. Future research could revise and supplement this study using methods such as cohort studies. It is recommended that future studies use a randomized controlled design to enhance the credibility of their findings.

## FUNDING

The subject of Department of Health of Hebei Province (CN): 20191827. The subject of Handan Science and Technology Bureau: 19422083009–33.

## CONFLICT OF INTEREST

All of the authors had no any personal, financial, commercial, or academic conflicts of interest separately.

### PEER REVIEW

The peer review history for this article is available at https://publons.com/publon/10.1002/brb3.2528

## Data Availability

All data generated or analyzed during this study are included in this published article.
